# Fabrication of nanofibrous mat surrounded hydrogel scaffold as an encapsulation device for encapsulating pancreas β cells

**DOI:** 10.1038/s41598-022-25736-8

**Published:** 2022-12-19

**Authors:** Mahdiyeh Sadat Seyyedi, Mohammad Monfared, Esmaeil Mirzaei, Negar Azarpira

**Affiliations:** 1 Student Research Committee, Shiraz University of Medical Sciences, Shiraz, Iran; 2 Department of Medical Nanotechnology, School of Advanced Technologies in Medicine, Tehran University of Medical Sciences, Tehran, Iran; 3 Department of Medical Nanotechnology, School of Advanced Medical Sciences and Technologies, Shiraz University of Medical Sciences, Shiraz, Iran; 4 Transplant Research Center, Shiraz University of Medical Sciences, Shiraz, Iran

**Keywords:** Biotechnology, Medical research, Nanoscience and technology

## Abstract

The main barriers to cells or organ transplantation such as pancreatic β-cells are the need for lifelong immune suppression and the shortage of donors. It may be overcome via cell encapsulation and transplantation techniques. Hydrogels provide a suitable ECM-like microenvironment for cells to adhere, survive, and function, while weakly performing as an immune barrier. In this study, we aimed to macro-encapsulate islet cells in a dual encapsulation device with collagen hydrogel and PCL nanofiber to provide an immune-isolated environment for cells to function more efficiently, where immune cells are not allowed to enter but oxygen, insulin, and nutrients can pass through. PCL thin mats with the pores diameter of 500 nm were synthesized by electrospinning and characterized by scanning electron microscope, porosity measurement, tensile strength test, and contact angle measurement. Collagen hydrogel was fabricated by extracting collagen fibers from rat tail tendons and solving them in acetic acid. β-cells (CRI-D2 cell line) encapsulated after neutralizing collagen solution (pH ≈ 7.4). Cell-collagen gel complex was poured into the nanofibrous mat packets to fabricate the whole device. Histology evaluation, cell viability, and cell function tests were done in 10 days. Live/dead assay of Cri-D2 cells encapsulated within the device showed that cells have diffuse distribution at the core of the hydrogel and the device. Also, cluster formation was seen and shows these cells can live in groups. To identify cells’ function within the device in these 10 days samples’ supernatant insulin level was measured by chemiluminescent immunoassay. It just showed a positive result for existing insulin within the medium. Based on our results, this device presents adequate features to be a good immune-isolation device for cell transplanting.

## Introduction

Many years of basic research have shed light on the value of regenerative medicine as a novel and powerful weapon against acute injuries or chronic diseases. Until now artificial organ transplantations, tissue grafts, and cell therapies have had significant progression; some of their products succeed to achieve FDA approval and many of them are still in the preclinical stages. Burn wounds, cartilage defects, corneal traumas, some types of hematopoietic cancers (e.g., B-cell lymphomas), neurodegenerative diseases (e.g., Parkinson's disease), hormonal dysfunctions (e.g. growth hormone deficiency, diabetes, etc.) and much other pathology can benefit from this interdisciplinary field^1–5^. Diabetes mellitus (DM) is a serious lifelong autoimmune disease characterized by hyperglycemia and low insulin level in the blood. It has two common types, T1DM and T2DM. Type 1 DM is associated with decreased insulin production due to the destruction of pancreatic beta cells (β-cells) by T-cell-mediated immunity^6^. DM is one of the important causes of morbidity and mortality, and also the cost of diabetes management for countries is large; hence, diabetes is a huge economic burden globally^6,7^. Although endeavors have been made for its treatment through these years, patients still face many difficulties and disease complications that affect their quality and quantity of life. Insulin therapy is a conventional treatment that has its pros and cons. Insulin therapy is almost always concomitant with episodes of hypo and hyperglycemia even with highly controlled insulin dosages while they never happened in non-diabetics in regard to state variable feedbacks^8–10^. In order to overcome this obstacle several alternative treatments have been developed, such as artificial pancreas^11^, pancreas transplantation^12^, stem cell therapy^13^ and islet transplantation^14^. Both pancreas transplantation and islet transplantation are now indicated for selected patients who deal with glycemic lability or hypoglycemic unawareness. Although solid organ transplantation is the current standard, islet transplantation is less invasive and associated with fewer risks while presenting similar results with whole organ transplantation^14,15^. Now, islet transplantation is done through the Edmonton protocol in that the allogeneic islets are directly infused into the portal vein after pre-transplantation processes^16,17^. With further evaluations and trials, islet transplantation challenges come to the light. The main barriers to its wider clinical applications are the need for lifelong immune suppression and the shortage of donors which may be overcome with cell encapsulation techniques and stem cell therapy, respectively^13,18,19^. However, islet transplantation combined with regulatory T cell immunotherapy is an alternative immunomodulatory approach, but they are not enough, and immunosuppressive is still needed^20^. For many years, hydrogels have been applied for cell encapsulation. They provide a suitable and extracellular matrix (ECM)-like microenvironment for cells to adhere, survive and function effectively^21^. Type I collagen, alginate, and gelatin are some examples of natural and synthetic scaffolds enjoyed in previous and current applications^22–24^. Even though hydrogels offer many advantages, they weakly perform as an immune barrier^25^. Encapsulation of transplanted cells with a semi-permeable membrane provides an immunoisolate environment for cells to function more efficiently where immune cells are not allowed to come in while oxygen, insulin, and nutrients can pass through^19^. Two strategies of cell encapsulation are available, microencapsulation (involving single cell encapsulation) and macroencapsulation (involving a cluster of cell encapsulation)^26^. Microencapsulating strategies provide maximum surface-to-volume ratio and nutrition exchange but restrict the control over membrane pore size and thickness^27^. On the other hand, macroencapsulation limits cell interactions with the environment, however; membrane parameters can be controlled well. In addition, macroencapsulation devices due to their large size could be transplanted and tracked in tissues more easily, even though this may result in foreign body reaction and fibrotic enclosure^28,29^. The biocompatibility of the biomaterial used in macro-encapsulation is principal since it can determine the probability of acute or chronic post-transplantation immune responses. Poly(ε-caprolactone) (PCL), chitosan, and polyurethane are among the appropriate options for this purpose. These polymers are also scalable in fiber arrangement, thickness, and pore size. Pore size regulation is important because other minuscule immune components like IgG, IgM, and complement mediators play a role in rejection besides immune cells^30,31^.

In this study, we aim to macroencapsulate islet cells in a dual encapsulation device with collagen hydrogel as the core and PCL nanofiber as its envelope. We chose collagen type I because it is approved for clinical applications due to its low antigenicity and low inflammatory response, plus its abundance in natural ECM, so, it can mimic cells' natural environment^32–34^. PCL is also an accepted biomaterial with biocompatibility and biodegradability that we do not need to remove it from the body; although it is biodegradable, it takes time to enzymatically hydrolyze so it is stable enough to properly play its role as a barrier. It is removed from the body through renal excretion and won’t accumulate in the body. The characteristics of the polymer make it an interest for electrospinning and tissue engineering^35–37^. Here, we assess the quality of hydrogel and nanofiber, and then evaluate in vitro cell survival and function (insulin production) within the device. Our prospect is to have transplanting devices selectively permeable to the nutrition and essential substances while inhibiting immune cells to pass through.

## Results and discussion

### Fabrication and characterization of PCL nanofibrous mats

**Figure 1:**
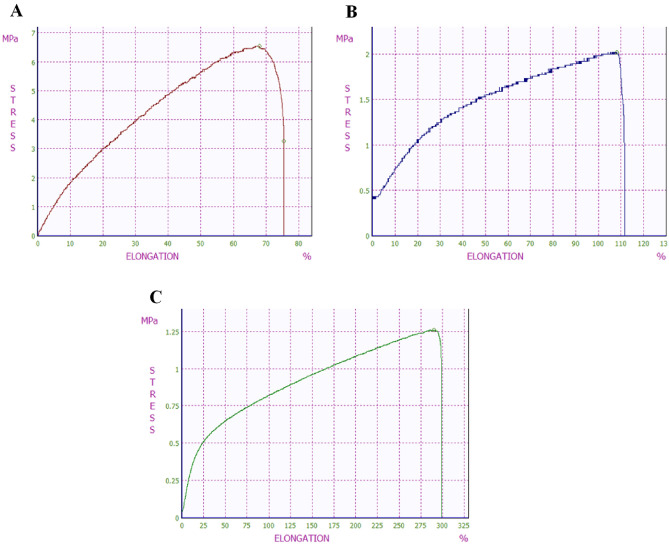
The tensile strength test charts of (**A**) 5%, (**B**) 7.5%, and (**C**) 10% PCL solutions.

To fabricate the PCL mats, we first electrospun nanofibers onto the rotating aluminum foil. Three concentrations of PCL solutions were used, 5%, 7.5%, and 10%. Choosing the best concentration is based on proper mechanical stability, sufficient hydrophobisity to avoid cell attachment, suitable fiber diameter to minimize the foreign body reaction (FBR) and proper pore size and porosity to ensure mass transfer without cell penetration. Determining the mechanical properties of the electrospun PCL nanofibrous mat, we placed the 10 × 30 mm mat between two grips and pulled them vertically at a speed of 10 mm/min until they tore. In this experiment peak force, tensile strength (maximum load the material can support without breakage), elastic strain, and Young’s modulus were calculated. Figure 1 shows the changes for each nanofibrous mat.

Tensile strengths were 6.53, 1.6, and 1.25 MPa for 5%, 7.5%, and 10% PCL solutions, respectively. Elastic strain is defined as the maximum elongation that the mat can withstand without permanent deformation. Young’s modulus also is a property of the mat that shows its elasticity; therefore, the higher young’s modulus indicates the more stiffness of the mat. Here, the nanofibrous mat made out of 5% PCL solution represents the highest Young’s modulus, 0.2 MPa. Throughout the experiment, the traction force was increased till the mat tore; this is called peak force. As presented in Table 1 the 5% PCL solution can tolerate 1.96 N before tearing, the highest peak force among other solutions.

**Table 1:** Tensile properties of PCL solutions with different concentrations. Although the elastic strain of the 5% PCL solution is relatively low, Young’s modulus is way higher than others.

PCL solutions	Peak force (N)	Tensile strength (MPa)	Elastic strain (%)	Young’s modulus^a^ (MPa)
5% PCL solution	1.96	6.53	10	0.2
7.5% PCL solution	0.99	1.6	25	0.03
10% PCL solution	0.61	1.25	25	0.02

^a^Young’s modulus: a measure of the ability of a material to resist changes in length when under tension or compression.

**Figure 2:**
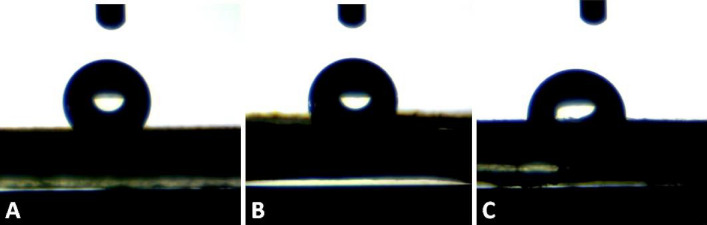
The surface contact angle of different PCL solutions in the 20th second (*P*-value < 0.0005). (**A**) PCL 5% (**B**) PCL 7.5% (**C**) PCL 10%.

The main drawback of using PCL in tissue engineering is its hydrophobicity; because it interferes with cell growth and proliferation^38^. In contrast, we define this characteristic as an advantage. Here, the goniometric measurements showed that the nanofibrous mat made of 5% (w/w) PCL solution represented a surface contact angle of 126 ± 1°, when the concentration of PCL increased to 7.5% and 10% the surface contact angle decreased to 103 ± 16° and 95 ± 9°, respectively (Fig. 2). These data show a significant difference between solutions (*P*-value < 0.0005). Superhydrophobic materials which represent limitations for protein adsorption, cell and bacterial interaction, are mainly described with their high apparent contact angle (i.e. exceeding 150°)^39^. Even though PCL is not identified as superhydrophobic, its high contact angle (especially the mat with 5% (w/w) PCL concentration) can have similar effects. These are necessary because our device would perform properly only if white blood cells and proteins in the blood circulation, especially ones associated with the immune system, do not attach to it. With this in mind, their attachment to the device surface covers the pores, initiates FBR and fibrosis, and then stops the mass transfer.

**Figure 3:**
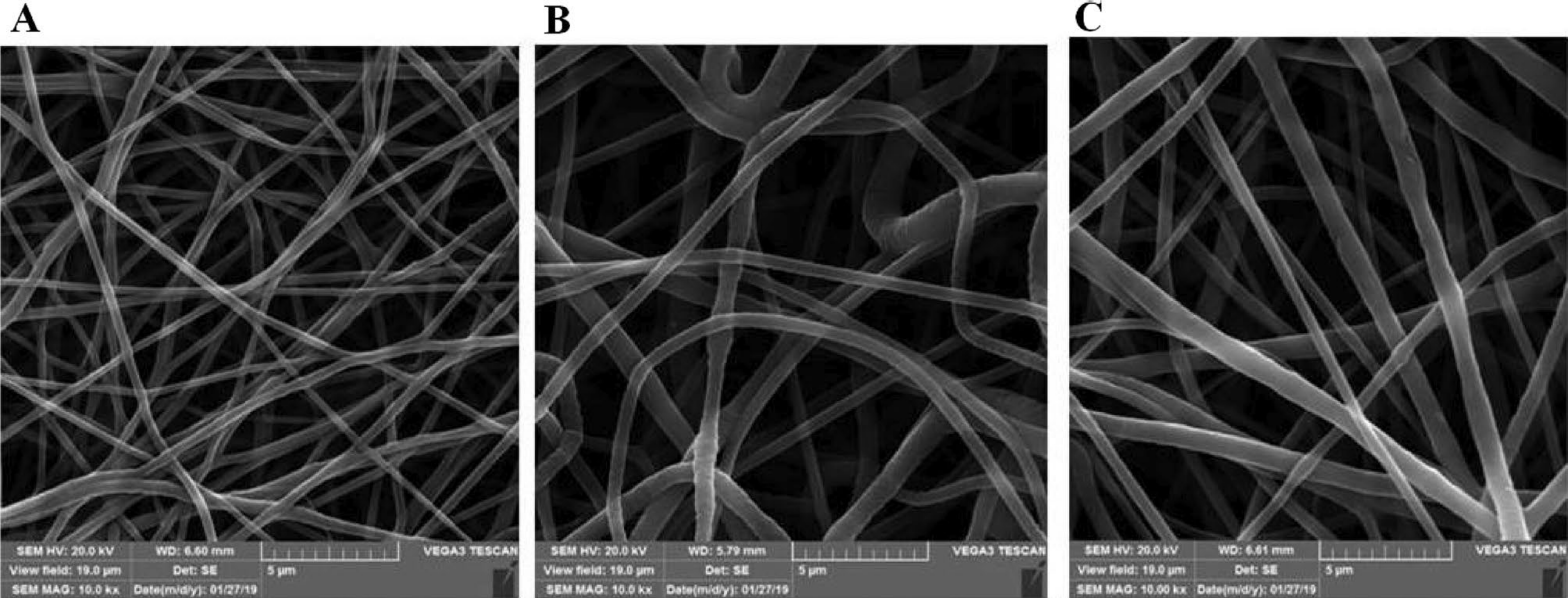
SEM image of different PCL nanofibrous mats obtained from (**A**) PCL 5%, fiber diameter of 254 ± 72 nm. (**B**) PCL 7.5%, fiber diameter 576 ± 238 nm, and (**C**) PCL 10%, fiber diameter 549 ± 217 nm. (Scale bars: 5 µm).

SEM images of PCL electrospun mats from the different concentrations of PCL solutions are shown in Fig. 3. All samples represented straight and smooth fibers without any defects or beads with a diameter in the range of 254–576 nm. The average fiber diameter of 5% PCL was 254 ± 72 nm. When the concentration of PCL solution increased to 7.5%, the mean fiber diameter increased to 576 ± 238 nm. However, for the 10% PCL solution, the mean fiber diameter was not changed remarkably. The 10% PCL showed a fiber diameter of 549 ± 217 nm. Because fiber diameter is associated with electrospinning parameters (e.g., voltage, flow rate, TCD, etc.), PCL concentration and solvents composition and ratio; our data are different from data that others obtained in their experiments. For instance, Nisbet et al.^40^ obtained nanofibers with 750 ± 100 nm diameter from 10% w/v PCL solution (same solvent as this study) applying 20 kV accelerating voltage. Ghosal et al.^41^ produced nanofibers from 8% w/v solution with wider diameter distribution, 0.4–2 µm, while utilizing 10 kV voltages. Moreover, Gluck^42^ reported that the 10 wt % PCL concentration (in comparison with 5 wt %) in the same solvent as us, is better for tissue engineering and produces fibers with a mean diameter of 426 ± 186 nm; however, the voltage applied in that experiment was 45 kV. Whereas, we demonstrate here 5 wt % PCL solution could be used for electrospinning and produced smooth and continuous fibers without forming beads even with narrower diameters. Fiber diameter not only can affect the pore size, but it can stimulate FBR initiation. As Wang et al.^43^ experiment show that the devices with fiber diameter below 500 nm had no cell penetration and induced minimal FBR and fibrosis.

**Figure 4:**
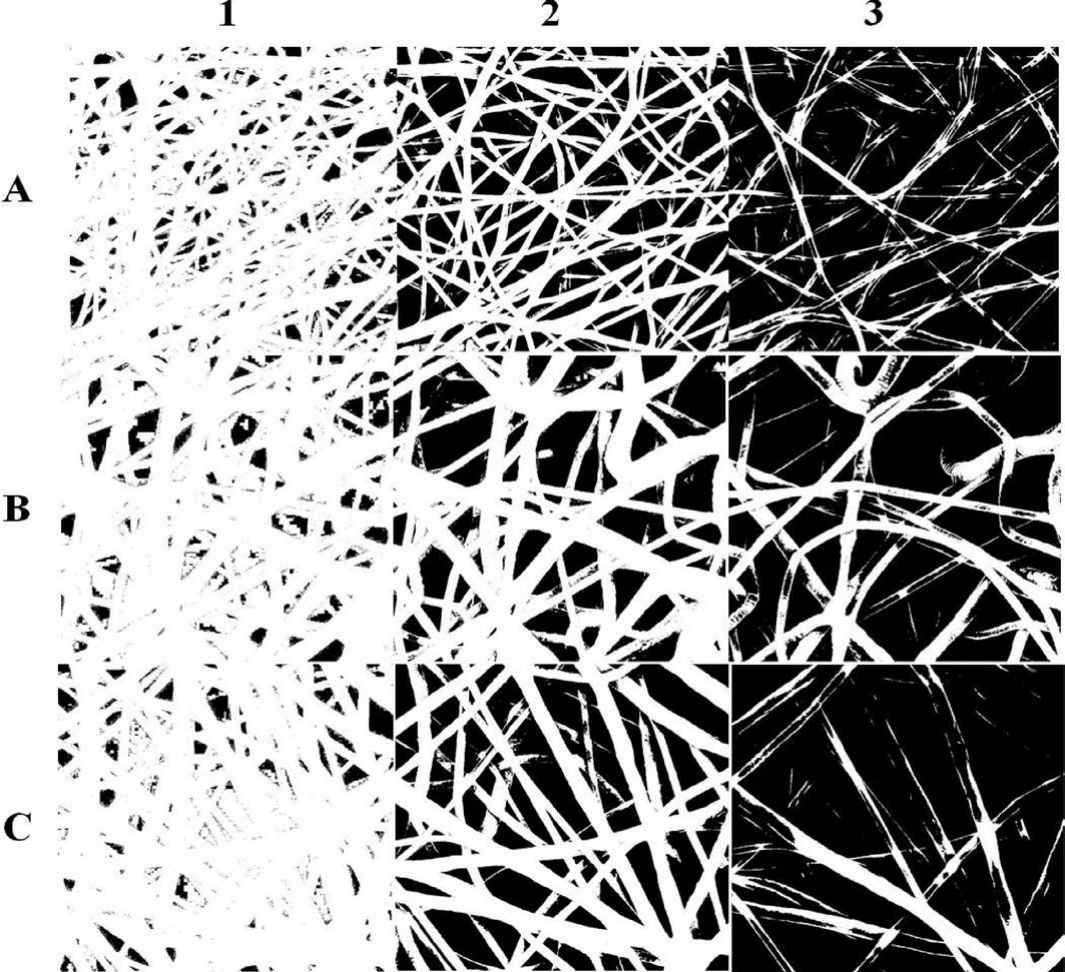
Nanofibrous mats different layers provided by the different thresholds of grayscale in Image J software. Rows (**A**), (**B**), and (**C**) are related to PCL 5%, 7.5%, and 10%, respectively. Columns 1, 2, and 3 are represented L1, L2, and L3 of nanofibrous mats, respectively.

Nanofibrous mats have different layers with different porosity and pore size. This occurs due to the overlapping of fibers at the deeper layers; therefore, a superficial layer contains a larger pore size with higher porosity than deep layers. This characteristic is very important in tissue engineering especially when we want to use them as filters or barriers (e.g. current study)^44^. As shown in Fig. 4, we separate each mat into three different layers and measure their pore size and porosity individually. This becomes possible by using different thresholds for each SEM image in ImageJ software.

In Table 2 the pore size and porosity of different samples are shown in detail. As we see the mat which is made of 5% wt PCL solution is the most porous among others for the deepest layer (L1). Besides, its L1 layer pore diameter (327 nm) is lower than mats made of 7.5% and 10% wt PCL solution. The most superficial layer (L3) as well, represents a suitable pore size (8.4 µm); while White Blood Cells (WBCs) including lymphocytes, natural killer cells (NK cells), and macrophages sizes are 10 µm, 12 µm and 21 µm, respectively^45–47^. Even Red Blood Cells (RBCs)—the smallest cell in blood circulation—with almost 7.5–8.7 µm in diameter^48^ would not trap in and obstruct surface pores of the device. It is obvious that angiogenesis is very important in tissue engineering and regenerative medicine; thus, pores should be large enough to allow capillary formation through them. The capillary diameter in the human body is about 8 µm which is almost the same size as the pore diameter in the L3 layer of our device^49^. Albeit, other minute components of the immune system including the complement system, cytokines, and chemokines (< 70 kDa) are much smaller than the device pores, so they can transmit easily through the device layers and reach the encapsulated cells^50^. In fact, the consequences of the interaction between cells and these tiny immune components and the response of the immune system to this interaction must evaluate further in in vivo studies and animal models. On the other hand, in Liu et al. experiment, when the nanofibrous device was coated with hydrogel it could avoid cell penetration and cell escape in pore sizes ranging from 0.15 to 1.67 µm; while cell escape and cell penetration were observed for the uncoated device with 1.67 µm pore size. So coating the device would boost its role as a barrier, while it would not disturb the mass transfer^51^.

**Table 2:** Pore size and porosity (percentage) of each sample with different layers. L1: the deepest layer, L2: the middle layer, and L3: the most superficial layer.

Samples	L1 Porosity/pore size		L2 Porosity/pore size		L3 Porosity/pore size		Mean porosity (%)
5% PCL	15%	327 nm	56%	1.4 µm	83%	8.4 µm	51.33
7.5% PCL	8.5%	445 nm	57%	3.7 µm	84%	10 µm	49.83
10% PCL	9%	431 nm	59%	4.8 µm	85%	23 µm	51

After all these tests on the nanofibrous mat made from different concentrations, the 5% PCL solution was selected for the rest of the experiments. Because mats electrospuned from PCL 5% solution represent beadless narrow fibers with low SD, proper pore size and porosity, and high tensile strength and young’ modulus. This mat also is the most hydrophobic among others.

### Cell encapsulation and proliferation

**Figure 5:**
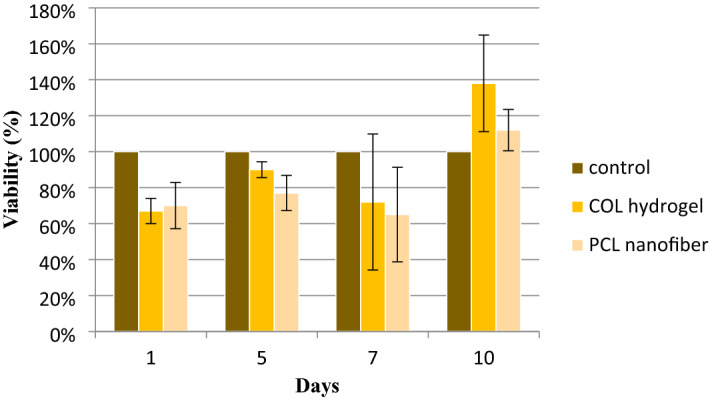
Viability of control, collagen hydrogel, and PCL nanofibrous mat groups on different days. Cell viability for hydrogel decreases on the 7th day while for PCL nanofibrous mat it decreases on the 5th day. There is no significant difference between the control and test groups. (*P*-value > 0.5).

To investigate our device’s potential for cell encapsulation and proliferation; we use the Cri-D2 cell line as a model. The toxicity of materials used in the encapsulated device is one of the main reasons which interfere with cell proliferation. PCL nanofibrous mat was examined by MTT assay to verify its cytotoxicity for Cri-D2 cells; however, FDA approved it for biomedical purposes. Also, we investigated our own fabricated hydrogel toxicity to confirm its safety; although collagen is the natural polymer and it is the most abundant protein in the ECM. We first chose 10^5^ cells for our experiments. Cri-D2 cells were cultured in a 24-well plate with a cell culture medium containing a piece of nanofibrous mat. In contrast, Cri-D2 cells were encapsulated in collagen hydrogel and then placed in a cell culture medium. Their viability presents in Fig. 5, shows PCL nanofibrous mat and collagen hydrogel are not toxic for these cells so they could proliferate and grow successfully. On the 10th day, the OD of both PCL and hydrogel are higher than the control group, it is due to the overexpansion of cells in control wells and lack of space in them. As a result, we halve the number of cells for further experiments (5 × 10^4^ cells for live/dead assays and insulin level). In another study, An et al.^52^ cultured 10^4^ in a 24-well plate and obtained high cell viability, but according to our results, with 5 × 10^4^ cells also high cell viability is observed. Here with 10^5^ cells, cell viability for the hydrogel group on days 1, 5, and 7 was 66%, 90%, and 72% and for the PCL group was 70%, 77%, and 65%, respectively. It can be concluded from cell viability percentages and Fig. 5 that cells survive and proliferate better in hydrogel than in PCL mat. This may be related to the hydrophobicity of the PCL mat that would not let cell attachment well. This is why we use collagen hydrogel in the core as a scaffold and utilize the PCL mat only as a barrier. According to the FDA standards, cell viability higher than 70% in cell-based products is permitted for clinical use^53^.

**Figure 6:**
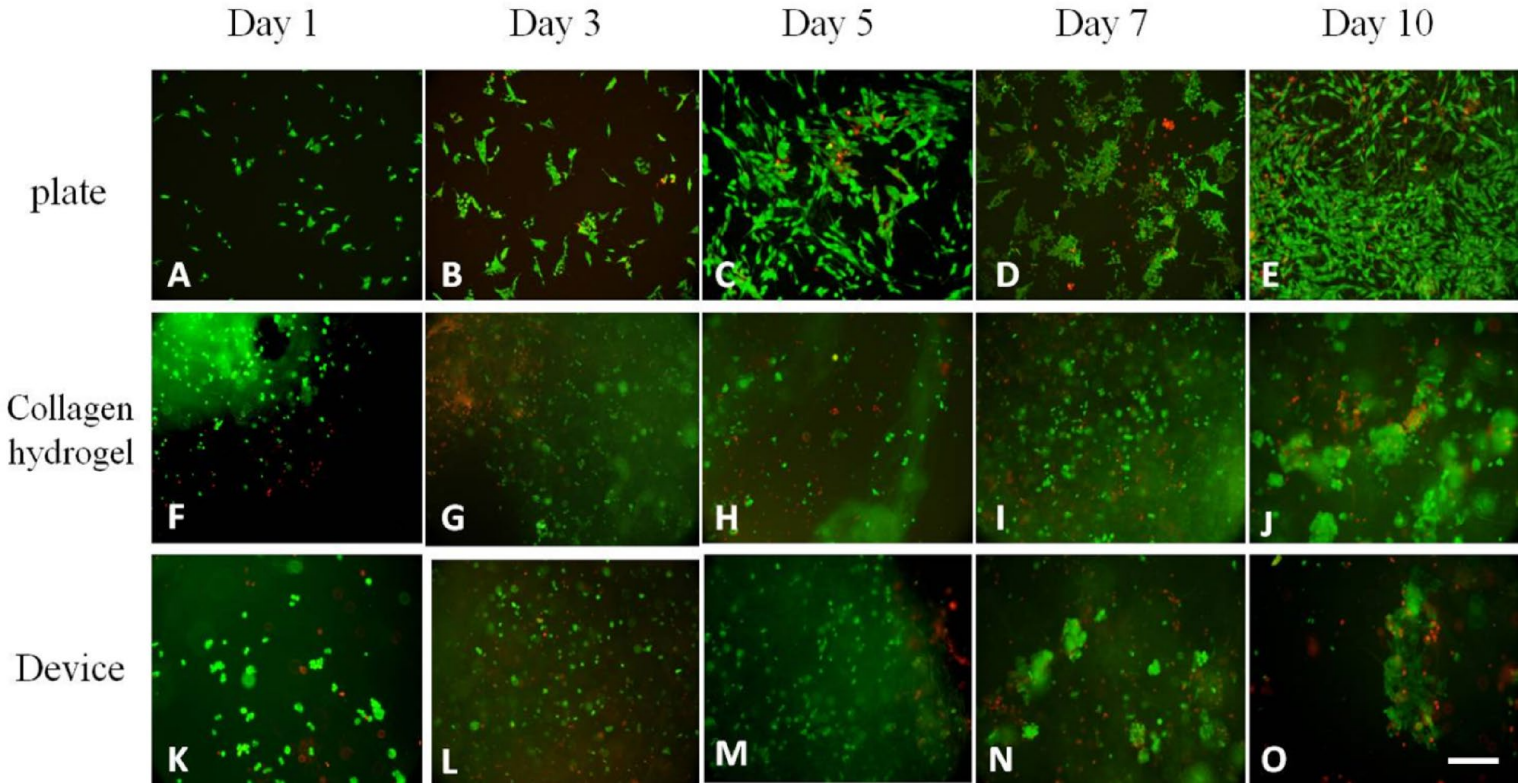
Live/dead assay of Cri-D2 cells encapsulated in collagen hydrogel, within the device and cultured in a 12-well plate. Cells have diffuse distribution and many green-stained cells are seen at the core of the hydrogel and device (**F**–**O**). Cluster formation (**I**, **J**, **N**, and **O**). The number of cells in plates (**A**, **B**, **C**, and **D**) are near to the cells in collagen hydrogel and the device; while many cells cultured in the plate died on the 10th day due to a lack of space to grow (**E**). (Scale bar: 200 µm).

**Figure 7:**
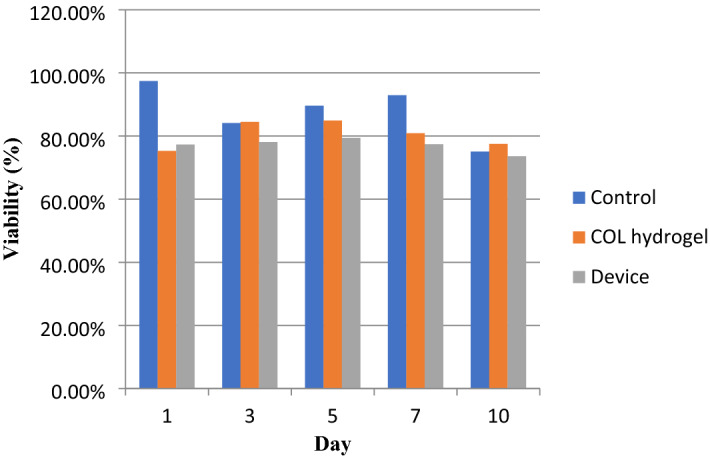
Cell viability in different groups. Generally, the Cri-D2 cells' viability in hydrogel and the device were hopeful for use in medical applications. The proliferation of cells in the hydrogel and device group increased by day 5 and then decreased by almost 75% by day 10.

The cell viability of Cri-D2 cells was estimated with live/dead double staining. Since we did not have access to a confocal microscope in our lab; we took several pictures with different focuses and then merged them together to make presented pictures (Fig. 6). This could show us the presence of cells in different layers of hydrogel mass. Then, these pictures were interpreted with Fiji software to obtain a cell viability chart. The high amount of green stained cells was approximately similar and represented a high ratio of live cells in all three groups. Cri-D2 cells showed about 75.4 ± 1.6% viability on the 10th day in all groups (Fig. 6E, J, O). This similarity in the percentage of viable cells on the 10th day in tests and control groups, in contrast with MTT results for the same day, may be due to decreasing the number of cells to 5 × 10^4^ in the live/dead staining test. The pattern of changes in viability through time almost followed the MTT assay results which showed cell viability increased until the 5th day and then decreased. The viability ratio in the device group compared to the hydrogel group was relatively lower while the process in which we extract the hydrogel bulk from the nanofibrous pocket was somehow aggressive and some cells may die in that process. As a result, the viability in the hydrogel group did not show any significant difference from the control group (*P*-value > 0.1), though the device group did (*P*-value < 0.05) (Fig. 7). The green and red stained cells are evenly distributed in the hydrogel and device which means oxygen and nutrients could efficiently reach the encapsulated cells. Some clusters of cells with different sizes could be seen with a close look at the cells encapsulated in the hydrogel and device, especially on the 7th and 10th day. This may represent that cells can live in groups in our dual encapsulation device. Thus, collagen hydrogel is a good scaffold for Cri-D2 cells and also the nanofibrous mat does not interfere with oxygen and nutrient passage.

### Cell function—insulin level

The supernatant of all samples in each group were collected in microtubes on day 1, 3, 5, 7 and 10, before performing live/dead assay. For those samples which their medium was replaced on certain days to mimic the blood circulation, we did not collect the previous medium and just use the last medium for testing. Insulin level was measured by chemiluminescent immunoassay. Because we encapsulated a relatively small number of cells in this experiment, the baseline insulin level was very low that the kit we use for measuring insulin level could not identify the quantity of insulin that existed in samples and just showed a positive result for existing insulin within the medium (≤ 0.184 µIU/mL). However, the results of our experiment would be more reliable if we perform the glucose-stimulated insulin secretion (GSIS) assay and use a more sensitive kit with a lower cut-off. As in An et al.^52^ study, although they use fewer insulin-producing cells (almost 20% of our cells), they measure much higher insulin levels due to GSIS assay. After all, this can demonstrate that Cri-D2 cells had a function while they were encapsulated within hydrogel or device through the period we conducted the experiment; but, long-term insulin level measurement also must be checked if we want our proposed device to be utilized in animal models.

### Hematoxylin & eosin (H&E) staining

**Figure 8:**
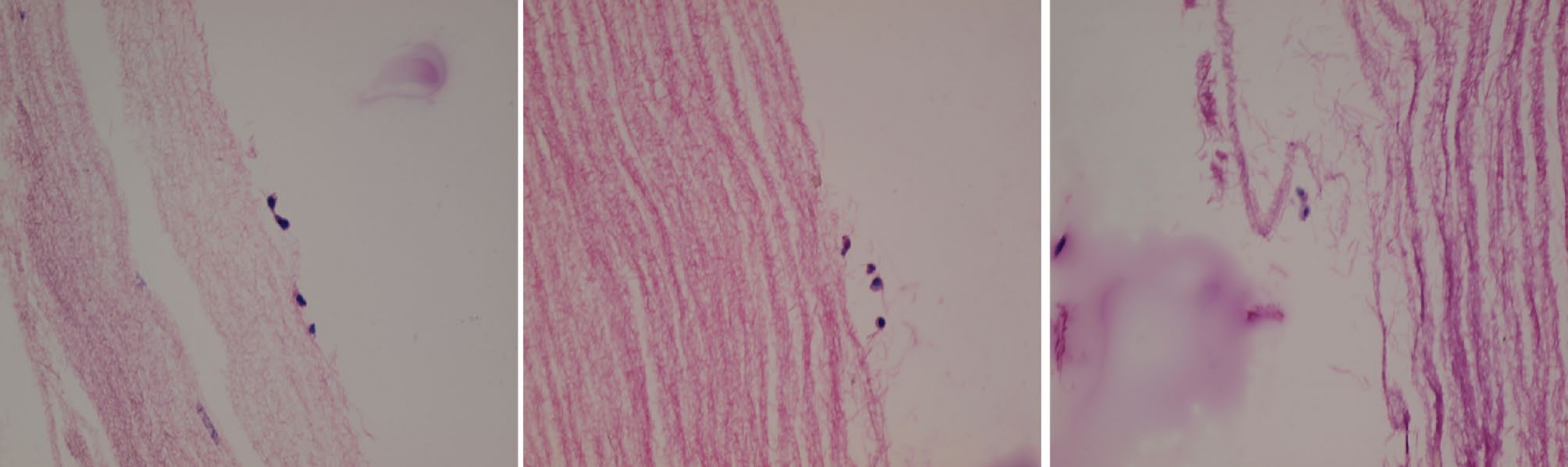
A device from 3rd day selected for (H&E) staining. Cells, nanofibrous mat (corrugated structure in pictures), and collagen hydrogel (soft structure) is seen.

The presence of cells was confirmed in H&E slides. Cells, collagen hydrogel, and nanofibrous mat is shown in the sections presented in Fig. 8.

## Conclusion

**Figure 9:**
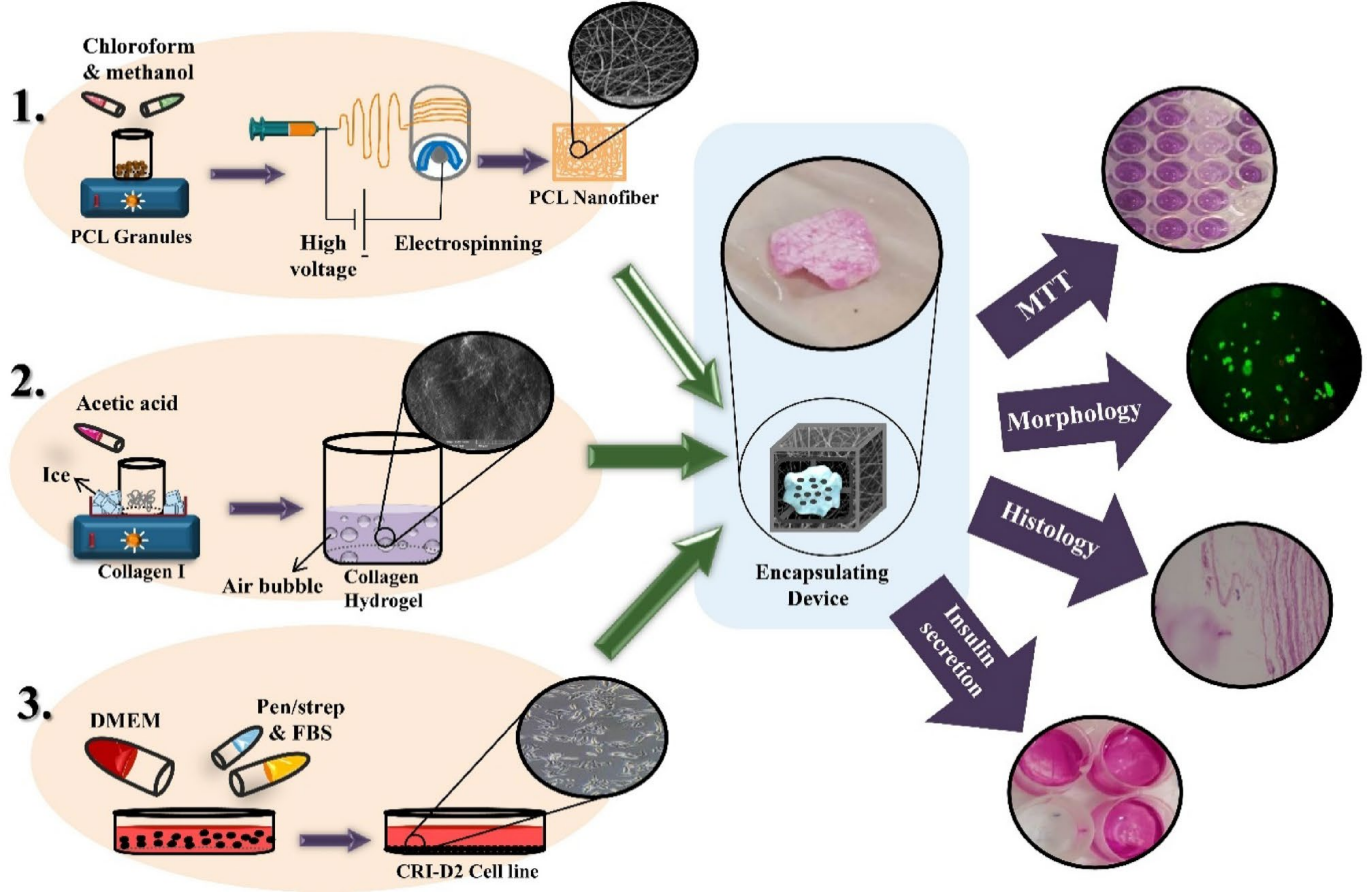
Collagen type I hydrogel is an acceptable scaffold and PCL nanofibrous mats have also demonstrated suitable characteristics as a membrane for β cells. therefore, cells can live and function while encapsulated in them.

Beyond the shadow of a doubt, fabricating an efficient device to overcome blood glucose fluctuation in diabetes strongly depends on finding a source of β cells and immunoisolation. Islets isolation from cadavers and stem cell-derived β cells (SC-β) are two sources for β cells with their own pros and cons^43^. In this study, we have shown that collagen type I hydrogel is an acceptable scaffold for β cells and they can live and function while encapsulated in it. PCL nanofibrous mats have also demonstrated suitable characteristics as a membrane. Although our fabricated PCL nanofibrous mat allows oxygen and nutrient passage, its function as an immune barrier must be studied in animal models (Fig. 9). It should be noted again that PCL is a degradable polymer and it must be used only for cadaver-isolated islets, not the SC-βs; while its degradation results in cell escape and teratoma formation by undifferentiated stem cells. Altogether, based on our results, this device presents adequate features, so examining it in further animal experiments would be reasonable. Even so, the device shape would be better changed for animal model studies. According to other studies, devices with sharp edges and rigid outer layers stimulate maximum FBR, in contrast with devices with smooth edges (i.e. tubular, cylindrical or spherical), soft outer membrane, and smaller sizes. As mentioned before, coating the nanofibrous device not only could decrease cell penetration and cell escape, but it provides a soft and smooth outer surface to minimize the FBR^43,51,54–56^. In the end, our suggested design for the device used in in vivo experiments would be a cylindrical nanofibrous wall coated with hydrogel, in a similar size to the current device (almost 1.5–2 cm in length).

## Method and materials

### Materials

Poly(ε-caprolactone) (PCL, average MW = 80,000) was obtained from Sigma-Aldrich chemical co. (Germany). Chloroform (CHCl_3_, ≥ 99%) and dimethyl sulfoxide (DMSO, 99.7%, cell culture–grade) were purchased from Merck Co (Germany). Fetal bovine serum (FBS), Dulbecco’s modified Eagle’s medium (DMEM Ham's high glucose), penicillin–streptomycin, trypsin–EDTA (0.25%), Phosphate buffered saline (PBS), and 2,5-diphenyltetrazolium bromide (MTT) were also bought from Sigma Aldrich co. (Germany). Cambridge Rat Islet-D2 (CRI-D2) cell line was obtained from Pasteur institute of Iran (Tehran, Iran).

### Nanofibers fabrication and characterization

Nanoporous PCL thin mats were made of three PCL mass solutions with different PCL concentrations by using ANSTCO RN-X electrospinning apparatus (Asian Nanostructures Co, Tehran, Iran). First, a mixture of methanol/chloroform in a ratio of 1:3 v/v was prepared. A combination of methanol and chloroform was used to obtain smooth, continuous, and beadless fibers while using chloroform as a solvent alone can result in beaded fibers due to its rapid rate of evaporation^57^. Then granules of PCL were added to the solvent mix while stirring with a magnetic stirrer at 70 °C to make 5%, 7.5%, and 10% (w/w) solution (in an impenetrable beaker). The solution was placed in a 10 mL syringe which had a blunted needle with an internal diameter of 0.8 mm. The syringe was attached to a syringe pump to control the flow rate. An aluminum foil was used as a fiber collector. A high voltage of 18 kV was applied to the needle tip; the flow rate and the tip-to-collector distance (TCD) were 1.5 mL/hr and 12 cm, respectively. All the experiments were done at room temperature.

Several analytic techniques were done for sample characterization. The morphology was observed by scanning electron microscope (SEM, TESCAN-Vega 3 (Czech Republic)) in magnification of up to 10000X. The porosity percentage and fiber diameter of the electrospun mats was determined by ImageJ software (n = 80). Uniaxial tensile tests were performed using the universal testing machine (SANTAM, Iran). Samples with a width of 10 mm were placed between two grips and stretched at the rate of 10 mm/min at room temperature. The wettability of fibers was also measured by contact angle (CA) measurements using an OCA 15 plus contact angle measurement system (Dataphysics, Germany) equipped with a CCD camera (precision ± 0.2°). The experiment was performed with 1 µL distilled water at room temperature. The water droplet was placed on nanofibers sheets within 1 min.

### Hydrogel fabrication

For collagen hydrogel preparation, twenty healthy male rats with a body weight of 195 ± 11 gr were euthanized in a cage filled with CO_2_. This procedure is based on the ARRIVE guidelines for how to handle animal subjects in research. Collagen fibers were extracted from rat tail tendons and collected in phosphate-buffered saline (PBS). All collected fibers were dispersed in 0.02 N acetic acid after further preparation and then well Stirred on a magnetic stirrer at 4 °C for 48 h. Next, the viscous solution was completely frozen at − 20 °C in the shape of small blocks. The frozen collagen blocks were put in a freeze-drier to lyophilize. Finally, the lyophilized sponge shape collagen was stored at − 80 °C until needed in the experiment. All methods in this study that have been carried out on animals have been in accordance with ethical guidelines for using animals in research. The ethical committee of Shiraz University of Medical Sciences has approved this with the ethical reference number, IR.SUMS.MED.REC.1401.444. To find detailed instructions on collagen extraction, please refer to Rajan et al.^58^.

16 g of collagen was added to 50 mL of 0.02 N acetic acid and well stirred on a magnetic stirrer at 4 °C until it was completely solved. The collagen solution was neutralized (pH ≈ 7.4) with 1 mM NaOH solution at 4 °C and then cells (CRI-D2 cell line) were added to the solution. The cell-collagen solution was pipetted into 15 wells and incubated for 15 min at 37 °C. After the gelation of the solution, 2.5 mL of cell culture medium was added to them. Characteristic tests of collagen hydrogel were done before and reported in another study^59^.

### Device creation

**Figure 10:**
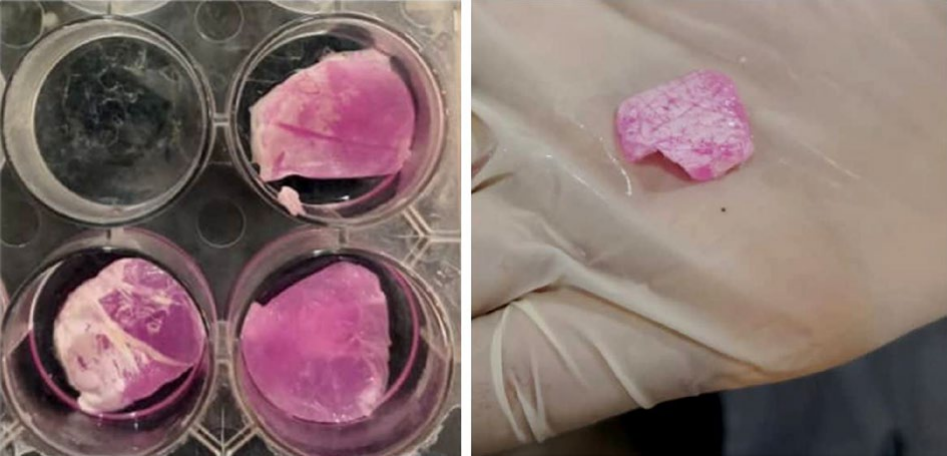
The cell encapsulation device. 1.5 × 1.5 cm nanofibrous packet containing cells and hydrogel.

At first, PCL thin mats were sterilized with 70% ethanol and subsequently placed under UV irradiation for about 2 h, before they were cut into smaller pieces and each piece was folded on itself. Two other edges were sealed with a sterilized thermal sealer machine to shape a 1.5 × 1.5 cm packet. Cell-collagen gel complex was poured into the packets and the open edge was closed immediately after filling. This step was done under the ice to prevent undesirable early hydrogel formation. The packets were put in a well of 12-well tissue culture plate, Cell culture medium was added and incubated at 37 °C in a 5% CO2 humidified atmosphere. Increasing the temperature to 37 °C cause fibrillation of collagen molecules and the formation of collagen hydrogel. Picture of the encapsulation device is shown in Fig. 10.

### Cell culture

Cells were seeded on a 75 mL flask and cultured in growth media (high-glucose DMEM with 10% fetal bovine serum (FBS), 1% penicillin/streptomycin) for 48 h at 5% CO_2_ and 37 °C. Cells were frozen and prior to use in experiments were defreezed. Cell counting was performed before each experiment by trypan blue dye exclusion method under an inverted microscope (Olympus/ Japan).

#### MTT assay

MTT assay to investigate CRI-D2 viability in collagen gel and PCL thin mat, the MTT assay was done after 1, 5, 7, and 10 days of cell seeding, and six different groups were defined;

1. Growth medium only (control for cells)
2. Growth medium with PCL thin mat (control for PCL)
3. Growth medium with collagen gel (control for hydrogel)
4. Growth medium with cells (control for two experimental groups)
5. Growth media, PCL thin mat and cells (experimental group)
6. Growth media, collagen gel and cells (experimental group)

All groups mentioned above were performed in triplicate in 24-well culture plate. PCL thin mats were cut into small pieces (15 mm in diameter) and placed at bottom of the wells for groups 2 and 5. Cell concentration was 10^5^ CRI-D2 cells/well. Cells were seeded in a 24-well culture plate for groups 4 and 5 (on the PCL thin mat), but for group 6 they were mixed with hydrogel and then pipetted into wells. 1.5 mL growth media was added to each well. To do the MTT assay, 150 µL media was replaced with 150 µL 3‐[4,5‐dimethyl‐2‐thiazolyl]‐ 2, 5‐diphenyl‐2H‐tetrazolium bromide (MTT) solution (0.5% w/v), and plates were incubated in 37 °C for 4 h. Later, the supernatant was completely removed and 300 µL dimethyl sulfoxide (DMSO) was added to dissolve formazan crystals and then distributed into 3 wells of a 96-well culture plate. 100 µL DMSO was used for the blank control group. Finally, their optical density was read by a microplate reader (Polar star omega, BMG LABTECH, Germany) at a wavelength of 570 nm. The below equation ${A}_{t}, {A}_{c}, {{A}_{cell} and A}_{0}$ represents the absorbance of hydrogel and PCL nanofiber test groups, control for each group, cell group and controlled DMSO, respectively.

$$cell\; viability\; percentage = \frac{{A_{t} - \left( {A_{c} + A_{0} } \right)}}{{A_{cell} - \left( {A_{c} + A_{0} } \right)}} \times 100$$

#### Survival tests (live/dead assay)

Cell viability was assessed using two fluorescent dyes, 0.01 mg/mL fluorescein diacetate (FDA) and 0.02 mg/mL propidium iodide (PI) which stain live cells and dead cells, respectively. Cells were mixed with dyes and incubated in the dark for about 60 s for only the cells group and about 120 s for the cell hydrogel and device group. It should be noted that the nanofibrous packet was cut and the hydrogel was extracted before staining. Then the sample was visualized under an inverted microscope (Olympus/Japan) using a blue light filter.

#### Function test (insulin level)

Cell culture media was removed and stored at − 20 °C, then the insulin secretion was evaluated by chemiluminescent immunoassay.

Experimental groups for cell survival test and cell function test (insulin secretion) are as followings:

1. Only CRI-D2 cells (control group)
2. Cell-hydrogel group
3. Device group

All three groups containing 5 × 10^4^ CRI-D2 cells and 1.5 mL high-glucose DMEM with 10% FBS. All experiments were performed in triplicate for each group and were followed for 10 days. Cell survival tests and cell function tests were done on days 1, 3, 5, 7, and 10.

### Histology evaluation (H&E staining)

Cellblock was prepared and fixed with formalin 10% and embedded in paraffin. Thin sections stained with hematoxylin and eosin (H&E).

## Data Availability

The data that support the findings of this study are available from the corresponding author, Prof. Dr. Azarpira, upon reasonable request.
